# Molecular Characterization, Via Next-Generation Sequencing, of Refractory or Resistant Invasive Breast Carcinoma

**DOI:** 10.7759/cureus.19528

**Published:** 2021-11-13

**Authors:** Patricia Pose Lapausa, Teresa Soria Comes, Inés Calabria, Inmaculada Maestu Maiques

**Affiliations:** 1 Surgical Pathology, Hospital General Universitario, Valencia, ESP; 2 Oncology, Hospital Universitario de la Ribera, Alzira, ESP; 3 Oncology, Imegen-Health in Code Group, Paterna, ESP; 4 Oncology, Hospital Universitario Doctor Peset, Valencia, ESP

**Keywords:** targeted therapy, precision medicine, molecular biomarker, next-generation sequencing, breast cancer

## Abstract

The most frequently diagnosed neoplasia in the world in 2020 was breast cancer (BC). On top of its high incidence, unexpected behavior as recurrence in patients, in spite of appropriate therapies, reaches 20%-30%. We believe that some molecular characteristics of tumors may lead to this bad behavior, and we can identify them with next-generation sequencing (NGS).

We made a retrospective multicentric study, conducted to molecularly characterize, by means of a custom NGS panel, cases diagnosed with treatment-refractory or treatment-resistant invasive breast carcinoma, studied in formalin-fixed paraffin-embedded (FFPE) samples. The panel included 50 genes related to tumorigenesis, cancer evolution and targeted therapies. Twelve cases were included from three centers. Alterations of driver genes were found in all of the cases, and 75% harbored mutations in TP53. Furthermore, we found alterations that could be therapeutic targets in half of the patients, such as mutations in PIK3CA (33% cases), mTOR (8.3%) or BRCA1 (8.3%). Other significant molecular alterations were: the loss of SWI-SNF complex´s components, modified genes of the MAP kinase pathway and alterations in epidermal growth factor receptor (EGFR). Not all of them are known targets but prognostic significance was found.

We conclude that NGS characterization of breast cancer in FFPE samples is a reproducible technique that can provide prognostic and predictive information about our patients and therefore, constitutes, in the near future, a valuable clinical tool in the context of precision medicine.

## Introduction

The most frequently diagnosed neoplasia in the world in 2020 was breast cancer (BC), and it has been estimated that it will also be the case for Spanish women in 2021. On top of its high incidence, recurrence in patients, in spite of appropriate therapies, reaches 20%-30% due to complex molecular mechanisms involved in its progression and resistance. This highlights the need for alternatives in patient management [1,2].

Next-generation sequencing (NGS) gives us the possibility to expand our molecular knowledge on this disease and better understand its pathogenesis. This technique consists of sequencing millions of DNA fragments in parallel, with a low cost per base. In oncology, it is particularly used to identify genetic drivers that can be further used for the identification of new therapeutic targets [3].

One of the main pioneers in this new era of cancer management was The Cancer Genome Atlas (TCGA). TCGA is a large-scale program that revolutionized cancer research by a systematic genome-based characterization of cancer. It began in 2006 and molecularly characterized more than 20,000 primary cancers, with up to 33 different types of cancer identified [4]. From it came many other programs that provide ideas and resources to help develop new cancer treatments. For example, Memorial Sloan Kettering Cancer Center from New York conducted an assay using formalin-fixed paraffin-embedded (FFPE) samples of solid tumors, sequencing selected exons and introns with a proprietary NGS panel, called Memorial Sloan Kettering-Integrated Mutation Profiling of Actionable Cancer Targets (MSK-IMPACT), to obtain the mutational profile for their actionable targets. After validation, they implemented it in their clinical laboratory to carry out prospective targeted sequencing and, thereby, guide therapeutic decisions [5].

The clinical impact of genomic analysis of the disease has already been recognized, thanks to the identification and use of diagnostic and prognostic biomarkers and those that are used as therapeutic targets. This is especially relevant in BC, because it is a heterogeneous disease in which targeted treatments are used in both localized and advanced [6].

We believe that some molecular characteristics of tumors may lead to unexpected behavior in patients receiving standard treatment. Therefore, the main objective of the present work was to molecularly characterize, by means of a custom NGS panel, a retrospective series of cases diagnosed with treatment-refractory or treatment-resistant invasive breast carcinoma to whom treatment was administered according to recommendations and clinical guidelines [7,8]. The secondary objectives were to evaluate the possible prognostic and predictive value of the observed mutations and to assess their possible clinical application in the context of precision medicine.

## Materials and methods

Study design

Patients for this descriptive, retrospective, multicenter study were selected from three centers in different healthcare areas of Valencia between January and June 2016. The same criteria for patient selection and the same clinical guidelines were followed at all three centers, and all cases were sent to the principal investigators for final evaluation.

Inclusion criteria were the following: patients of any sex and age, with invasive breast carcinoma refractory or resistant to therapy indicated by international recommendations, i.e., in which the tumor grows or spreads during treatment or progresses after therapy. In addition, there had to be a sufficient amount of sample available for study (≥ 6 mm^2^ of tumor sample).

The study was reviewed and approved by the hospitals' research ethics committee.

Patients whose relapse did not meet the criteria for resistance or refractoriness, those with immunodeficiencies or severe comorbidities that could cause a great acceleration of the natural history of the course of their cancer, those who had not undergone a radical/almost radical intervention (R0/R1) or those whose tumor sample was insufficient were excluded. Patients who did not attend their scheduled treatment were not considered. These features were evaluated by the responsible clinician and reassessed by the investigators of this research.

Overall survival (OS) was defined from the date of the first treatment to the date of death of any cause; and was estimated with a multivariate Cox regression model including biological subtype (triple-negative vs luminal) and stage. Statistical analysis was performed using the G-STAT software.

Pathology report

The following variables were considered:

- Nottingham histologic grade, which assigns a value to three features (gland formation, nuclear grade, and mitotic count) to obtain a total score that allows classifying the cancer's differentiation stage into three grades.

- Immunohistochemically defined intrinsic subtype, following the 2013 St Gallen Consensus criteria:

Luminal A (LA): estrogen receptor (ER)+, progesterone receptor (PR) >20%, human epidermal growth factor receptor (HER2) negative and marker of proliferation Ki67 (KI67) <20% 

Luminal B (LB): ER+, HER2-negative, PR <20% or KI67 >20% 

HER2-enriched

Triple-negative (TN): ER-, PR-, HER2-

- TNM classification 8th edition: T for the extent of the primary tumor, N for lymph node involvement and M for metastatic disease.

NGS panel 

According to the clinical guidelines and scientific literature from the time period in which the cases were collected, the panel (Action OncoKitDX, Imegen-Health in Code Group) studied the most relevant genes in adult solid tumors. Many of the genes were considered actionable according to the NCCN and the SEOM (National Comprehensive Cancer Network; Sociedad Española de Oncología Médica) or were related to treatments approved by the FDA or EMA (Food and Drug Administration; European Medicines Agency), as well as genes related to tumor genesis and potential therapeutic targets that were being tested in clinical trials.

The panel was used to sequence the complete coding regions of the following 50 genes: AKT1, AKT2, AKT3, ALK, ARID1A, ATRX, BRAF, BRCA1, BRCA2, CDH1, CTNNB1, EGFR, ERBB2/HER2, ESR1, ESR2, FGFR1, FGFR2, FGFR3, FGFR4, GNA11, GNAQ, HIST1H3H, HRAS, IDH1, IDH2, KIT, KRAS, MAP2K1, MET, MTOR, MYC, MYCN, NRAS, NTRK1, NTRK2, NTRK3, PDGFRA, PIK3CA, PBRM1, PMS2, PTEN, POLD1, POLE, RET, ROS1, SDHA, SDHB, TERT, TP53 and VHL. It was also used to test for fusions of the ALK, EGFR, NTRK1, ROS1, BRAF, RET, ETV6, NTRK2 and BCR genes with any other gene, CNVs in the whole genome and microsatellite instability (MSI) by testing for 110 biomarkers.

Sample processing

Matched normal and FFPE tumor samples were selected among the surgical specimens received at the Surgical Pathology Unit of each of the three participating hospitals for sequencing with NGS to identify somatic and germline genomic alterations. For all samples, the sequencing process was performed in the same laboratory and the analysis was carried out under the same conditions.

Laboratory work 

1. DNA extraction and quality control

Genomic DNA (gDNA) of the tumor and of the control tissue was extracted using the commercial extraction kits RecoverAll™ Total Nucleic Acid Isolation Kit for FFPE (Thermo Fisher Scientific, Waltham, MA, USA) and QIAamp DNA Investigator Kit (Qiagen).

Concentration was measured on a Qubit fluorometer (Thermo Fisher Scientific) employing Qubit dsDNA BR and Qubit dsDNA HS assay kits (Invitrogen). DNA integrity (DIN) was determined by DNA ScreenTape analysis (Agilent Technologies). 

2. Library preparation and sequencing

The NGS panel was based on Agilent's SureSelect XT HS technology and was designed to detect variants with low allele frequencies in small amounts of DNA from FFPE tissue samples.

Library preparation followed the instructions for use of Action OncoKitDx (Imegen-Health in Code Group). The libraries were validated and loaded on a NextSeq 550 sequencer (Illumina) for a 2x75 paired-end run set up according to the manufacturer's instructions.

3. Bioinformatics analysis

The whole bioinformatics analysis, including alignment with the reference sequence Genome Reference Consortium Human Build 37 (GRCh37, hg19), annotation and variant calls, was performed on the DataGenomics platform (Imegen - Health in Code Group). 

4. Functional classification of variants

Variants have been classified as pathogenic, probably pathogenic, variants of uncertain significance, probably benign and benign, according to the American College of Medical Genetics and Genomics (ACMG) guidelines [9]. Evidence for pathogenicity was supported by the COSMIC (Catalogue Of Somatic Mutations In Cancer), ClinVar, gnomAD and Varsome databases, clinical guidelines, such as NCCN and ESMO (European Society for Medical Oncology), clinical tests and scientific literature.

## Results

In the considered period, the cases that could be included were 12 women with invasive ductal carcinoma (IDC). During the follow-up of the study, all patients were deceased.

Table 1 summarizes the most important clinical and tumor characteristics, reason for inclusion, time at which the sequencing was performed in relation to systemic therapy and the treatment during which relapse or resistance occurred.

**Table 1:** Characteristics. P: patient; ST: subtype; NHG: Nottingham histologic grade; MIT: main reason for inclusion in the study; ET: endocrine therapy; neo-CTx: neoadjuvant chemotherapy (CTx); TS: time when the material was sequenced in relation to first-line or neoadjuvant systemic treatment; T-RR: therapy during which relapse or resistance occurred.

P	AGE	ST	NHG	TNM	MIS	TS	T-RR	SUBTYPE CHANGE AFTER CTX
No. 1	45	LA	2	T2N0M0	Progression during HT	Before treatment	ET: Tamoxifen	
No. 2	76	LA	2	T2N1M1	Progression during HT	Before treatment	ET: Palbociclib-Letrozole	
No. 3	37	LA	1	T2N1M0	Progression during HT	After treatment	ET: Palbociclib-Letrozole	
No. 4	44	LB	3	T2N0M0	Refractory to neo-CTx	After CTx		DX after CTx: TN subtype
No. 5	44	LB	3	T2N1M0	Refractory to neo-CTx	After CTx		
No. 6	38	LB	2	T2N0M0	Progression during HT	Before treatment	ET: Palbociclib-Letrozole	
No. 7	42	LB	3	T2N0M0	Refractory to neo-CTx	After CTx		DX after CTx: TN subtype
No. 8	44	TN	3	T2N0M0	Refractory to neo-CTx	After CTx		
No. 9	43	TN	1	T3N1micM0	Refractory to neo-CTx	After CTx		
No. 10	36	TN	3	T2N1M0	Refractory to neo-CTx	After CTx		
No. 11	69	TN	3	T2N1M0	Refractory to neo-CTx	After CTx		
No. 12	51	TN	2	T4bN3M1	Refractory to neo-CTx	After CTx		

After DNA sequencing, the observed results were single-nucleotide variants (SNV) and copy number variants (CNV), all of somatic origin. SNVs were defined by the chromosome they were located in, the position of the nitrogenous base, their allele frequency, the variant in the transcript and the positions in the cDNA and the protein, as well as their functional classification according to ACMG (Table 2). 

**Table 2:** SNV description. P: patient; VAF: variant allele frequency; FC: functional classification; SNV: single-nucleotide variants.

	GENE WITH SNV	POSITION	VARIANT	VAF	FC
P1	PIK3CA	3:178936091	NM_006218.2 c.1633G>A p.Glu545Lys	42% (136X)	Pathogenic
P2	PIK3CA	3:178952085	NM_006218.2 c.3140A>G p.His1047Arg	36% (129X)	Pathogenic
P3	PIK3CA	3:178952085	NM_006218.2 c.3140A>G p.His1047Arg	47.3% (205X)	Pathogenic
P4	BRCA1	17:41228617	NM_007294.3 c.4372C>T p.Gln1458Ter	69.9% (522X)	Pathogenic
	TP53	17:7577109	NM_000546.5 c.829T>G p.Cys277Gly	64.4% (153X)	Pathogenic
P5	mTOR	1:11167549	NM_006218.2 c.7643T>A p.Phe2548Tyr	28.3% (106X)	Uncertain significance
	TP53	17:7576853	NM_000546.5 c.993G>C p.Gln331His	67.4% (175X)	Pathogenic
P6	ARID1A	1:27101195	NM_006015.4 c.4477C>T p.Gln1493Ter	9% (204X)	Pathogenic
P7	PIK3CA	3:178952085	NM_006218.2 c.3140A>G p.His1047Arg	47.3% (205X)	Pathogenic
	TP53	17:7578190	NM_000546.5 c.659A>G p.Tyr220Cys	64.55% (110X)	Pathogenic
P8	TP53	17:7577099	NM_000546.5 c.839G>C p.Arg280Thr	71.1% (246X)	Pathogenic
P9	TP53	17:7574021	NM_000546.5 c.1004_1006delinsT p.Arg335LeufsTer11	16.56% (163x)	Pathogenic
P10	TP53	17:7577121	NM_000546.5 c.817C>T p.Arg273Cys	48.35% (393X)	Pathogenic
P11	TP53	17:7578394	NM_000546.5 c.536A>G p.His179Arg	76.61% (124X)	Pathogenic
	HRAS	11:534286	NM_005343.2 c.37G>C p.Gly13Arg	48.28% (203X)	Pathogenic
	PTEN	10:89685287	NM_000314.4 c.182A>T p.His61Leu	29.33% (75%)	Pathogenic
P12	TP53	17:7577121	NM_000546.5 c.817C>T p.Arg273Cys	48.35% (393X)	Pathogenic

Normalized read count was used for CNV analysis. Quality of the CNV calls was calculated on the basis of parameters such as log2 ratio, size of the event, proximity and contiguous events. Chromosomal gains and losses with different numbers of copies were detected in seven patients, including focal ones, of chromosomal arms and of complete chromosomes. CNVs that have been considered to be the most relevant are shown in Table 3.

**Table 3:** CNVs suggested by the bioinformatics analysis based on sequencing results. CNVs: copy number variants.

PATIENT	GENES AND TYPE OF CNV
P1	PBMR1: Complete loss of the gene
P2	MYC, FGFR1, BRAF, MET: Partial gain HIST1H3H: Complete gain ATRX: Complete loss
P3	TP53: Complete loss
P6	FGFR1: Amplification
P7	EGFR (HER1): Amplification
P8	TP53: Loss
P9	5q, 8p, X: Loss 8q, 10p: Gain

Alterations in driver genes were identified in 100% of the samples, with TP53 alterations observed in 75% of the patients. The most relevant actionable alterations were observed in the PIK3CA gene in 33.3% of patients (three LA and one LB patient); in the mTOR gene in 8.3%, comprising one LB case; and PTEN dysfunction in 8.3%, corresponding to one TN case. Gene fusions, microsatellite instability or germline alterations have not been identified in any of the cases. Alterations already reported in the literature as therapeutic targets and with approved targeted therapy were found in 50% of the patients.

During the five-year follow-up of the study, all patients were deceased. Two patients died because of causes different from cancer and their data was censored in the survival analysis. Also, only two of the patients were diagnosed of stage IV disease, and their survival is presented separately. The analyses of cause-specific OS for patients without metastases showed a 58-month median OS for cases with luminal cancer, and 16-month median OS for cases with TN disease. On the other hand, of the two patients with metastases, patient number two (LA) had a survival of nine months, and patient number 12 (TN) had a survival of 23 months. 

## Discussion

**Figure 1:**
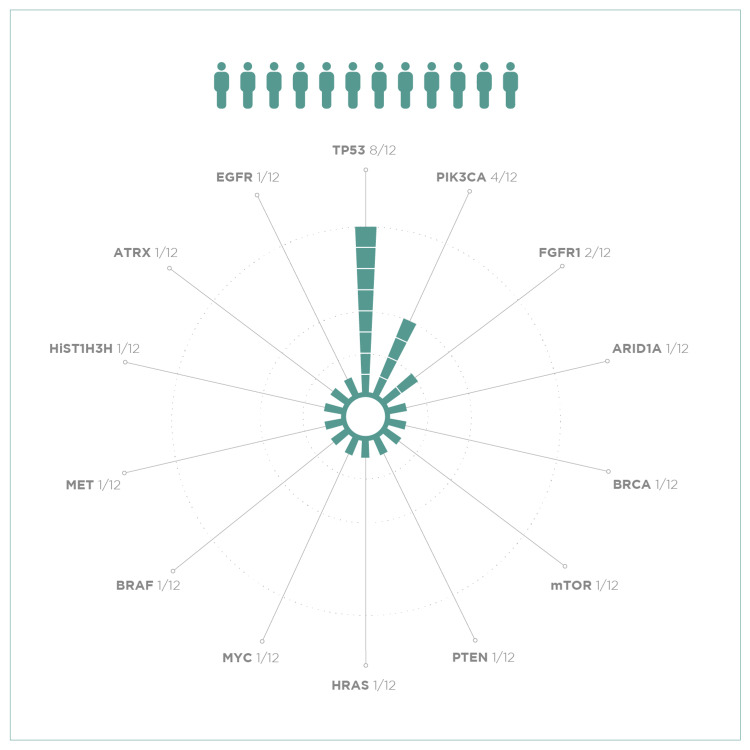
Genes in which alterations were seen in our work.

The present study describes somatic mutations detected by NGS using a custom panel in a series of 12 cases of invasive ductal BC resistant or refractory to systemic therapy. The mutations obtained were located in the TP53, PIK3CA, PTEN and ARID1A genes, among others (Figure 1). They correlate with the most frequently mutated genes in pan-gyn tumors (gynecologic and breast cancer) identified by Berger et al. as part of the TCGA Pan-Cancer Atlas Project [10].

Eight cases harbored variants in the TP53 gene, which totaled to 66.7% of the presented series. These patients relapsed early after-or during-treatment and who, therefore, constituted a group with a poor prognosis. TP53 is a tumor suppressor gene, known as "the guardian of the genome", and mutations in this gene are one of the most frequent mutations in BC, being identified in almost 30% of cases [11]. The literature shows contradictory results regarding the clinical relevance of its mutated state, since it has been associated with negative, positive and neutral results, so it is still a subject of debate [11]. Therefore, it does not seem that the aggressive course observed in our patients can be explained solely by an alteration in this gene.

A high percentage of patients included in this study (50%) had an alteration in the PTEN-PI3K-AKT-mTOR pathway. This pathway has a fundamental role in cell proliferation, growth and survival, being a biomarker with a poor prognosis which may have participated in the behavior of the cases that presented it [12]. In particular, activating variants in the PIK3CA and mTOR oncogenes were detected in 33.3% (three LAs and one LB that resulted in TN in the surgical specimen) and 8.3% (one LB), respectively, and variants inhibiting the function of the tumor suppressor gene PTEN in 8.3% (one TN). It is especially relevant that PIK3CA and mTOR are predictive biomarkers of resistance to various treatments: PIK3CA to endocrine therapy (ET) in BC with positive hormone receptor (HR), to CTx in general, and to anti-HER2 therapies in HER2-positive carcinomas, and mTOR to multiple drugs, particularly to tamoxifen. They are so relevant that the FDA and the EMA approved the use of the PI3K inhibitor, alpelisib, in combination with fulvestrant in postmenopausal women and men with ER+, HER2-, metastatic or advanced BC who are refractory to ET and have an alteration in the PIK3CA gene (SOLAR-1 study); as well as the use of everolimus, an mTOR inhibitor, in combination with exemestane (an aromatase inhibitor) in postmenopausal patients with luminal or metastatic BC refractory to treatment with aromatase inhibitors (BOLERO-2 trial) [6,13]. Following these criteria, patient 2, a 76-year-old woman with luminal carcinoma that had been refractory to ET and metastatic would have been a candidate to receive targeted therapy with alpelisib, which would justify having previously studied the status of the PIK3CA gene.

Similarly, given that PTEN is the main inhibitor of the PI3K pathway, it could be argued that patient 11, with PTEN dysfunction, could also benefit from treatment with inhibitors of the pathway. Nevertheless, there are teams that have already evaluated mTOR and AKT inhibitors for this purpose, and not enough evidence has been found to recommend a specific drug in the presence of a PTEN mutation [14]. 

On the other hand, 16.7% of our cases were found to have an alteration in genes that encode proteins of the SWI-SNF chromatin remodeling complex. Complete loss of the gene encoding PBRM1 was identified in patient 1, and patient 6 had an SNV in the ARID1A gene. It has been reported that approximately 20% of all human tumors contain alterations in this complex, and the gene that mutates most frequently in ER+ BC isARID1A [15,16]. Patient 6, who had subtype LB, had an ARID1A-inactivating mutation. Loss of function of this kind has been associated with resistance to ET and poor disease-free survival [16]. This patient had an extremely rapid evolution, progressed to treatment with CTX-RT and progressed again with the combination of palbociclib-fulvestrant, which could be explained by findings described above and would be in line with what was reported in a 2020 study on the loss of ARID1A function and resistance to the ER degrader fulvestrant [16]. On the other hand, genomic instability associated with defects in some SWI-SNF subunits, such as ARID1A or PBMR1, suggests a greater generation of neoantigens by carrier cells, indicating that these tumors are better targets for immunotherapy. However, prior knowledge of this alteration may enable a different therapeutic plan. Continuing with the SWI-SNF complex, the loss of PBMR1 might have participated in the poor outcome of patient 1, as it is considered a possible prognostic biomarker in BC associated with poor clinical results [15].

Alterations were also found in genes of the mitogen-activated protein kinase (MAPK) pathway. Patient 2 was found to harbor a partial gain of BRAF, and patient 11 had an SNV in HRAS. This pathway plays an important role in the pathophysiology of BC, and its persistent activation has been associated with the growth and metastatic activity of this cancer [17]. In turn, mutations in RAS and RAF family members have been reported to be very rare events in BC [18,19]. According to COSMIC, the frequency of mutations in RAS is less than 1% [18]. Furthermore, it has been described that a mutation in a member of the RAS family leads to constitutive activation of some of the pathways it modulates, such as the PI3K pathway, already altered in patient 11 due to PTEN dysfunction, suggesting that both alterations might have participated in the irregular evolution of the case. Their therapeutic potential is still under investigation [17].

In the case of RAF genes, non-V600E BRAF mutations have not been extensively studied. There is a 2020 study that examined the association of the BRAF/MEK pathway with the risk of breast cancer recurrence. It proposed that this pathway could be a biomarker of recurrence based on ER status, which would suggest its implication in the relapse of patient 2 [19]. This patient also had a partial gain of MYC and MET, and these variants, in line with information in the literature, where these variants are classified as biomarkers with a poor prognosis, would support the poor evolution of the patient [20]. The MYC amplification could explain the patient's resistance to HT, in this case to letrozole (an aromatase inhibitor), which was administered in combination with palbociclib [20]. As there is a treatment that targets MYC, it would appear as the logical strategy, but different studies emphasize its ineffectiveness in these cases. Instead, one could reflect on the role that the receptor tyrosine kinase (RTK) encoded by the MET gene, proposed in recent works as a potential therapeutic target in advanced BC, could have [21,22].

There are also other relatively frequent genetic alterations in BC, though they have not reached clinical practice, at least not yet. For example, EGFR overexpression, which is associated with poor clinical outcome, is described in 15%-30% of BC cases [23], but in this study, which included patients with a poor outcome, it was only observed in one LB case, whose surgical specimen was diagnosed as TN. Despite the existence of anti-EGFR therapy, it would not have been used in this patient, because clinical trials have shown disappointing results in triple-negative BC. But, for future cases, it would be convenient to bear in mind that some teams have reported that the efficacy of such therapy would increase if anti-RTK monoclonal antibodies were combined with immune checkpoint inhibitors, such as PD1 or CTLA-4 [23].

Genetic aberrations in FGFR are also frequent in BC. The literature shows FGFR1 amplification has been observed in 15% of HR+ carcinoma cases [24]. Patient 6 had a FGFR1 amplification, which could explain the rapid evolution and development of metastatic disease, caused by lack of response to ET, since it acts as a prognostic and predictive biomarker [24]. Patient 2 had a partial gain of the gene, without reaching amplification, which would require confirmation with MLPA (multiplex ligation-dependent probe amplification), the standard technique for CNVs [25]. In the near future, FGFR testing will open the possibility of targeted therapy [24].

Finally, the detection of a mutation in susceptibility genes for BC, such as BRCA, must be highlighted, since they constitute a prognostic factor, a predictive factor for treatment with targeted therapies, and, when found in the germline, a risk factor for the development of different neoplasms [26]. In our sample, only one case was found to have an alteration in the BRCA1 gene, an LB patient whose surgical specimen was diagnosed as TN. She did not meet the criteria for receiving the approved targeted therapy, but she did meet the criteria for prophylactic mastectomy of the contralateral breast [26].

On the other hand, the median cause-specific OS of the patients with local and regional disease was lower than that described in the previous publications: median OS of patients in our study with early breast cancer was lower than five years; however, European guidelines report that 10-year OS is around 70% in luminal early breast cancer [7]. Regarding TN disease, three-year OS is around 74%; however, median OS in our study is no longer than 16 months [27]. Although survival of patients with advanced disease is also lower in our investigation, conclusions cannot be drawn because only two patients with two different biological subtypes of breast cancer could be analysed. However, globally these results evidence a worse prognosis of the patients in our study compared to prior studies. 

Regarding the limitations of the present study, in the first place, it should be taken into account that sequencing of 75% of the cases was carried out on material that had received systemic treatment, which was probably a bias in this study because there is a possibility that it would be a source of enrichment with clonal mutations, or that the surviving clones have been able to execute an adaptive mechanism driving their resistance and tumor heterogeneity [28]. Though, it is true that there are no relevant studies that would have thoroughly analyzed the results of sequencing pre- and post-systemic treatment. For example, a Japanese group reported that all patients in their study had received surgical treatment, whereas the MD-ANDERSON working group carried out a trial in which all patients had failed conventional systemic treatment, to compare it with results after administering therapy directed at a molecular target [29,30]. Tumor heterogeneity could also be a bias at the time of choosing areas of the surgical piece for sequencing, but there are already teams that have evaluated the viability of molecular studies in small biopsies (<1.5 mm), and they have concluded that there is a correlation with results on large surgical specimens [31]. 

The NGS sequencing itself could have been one of the biases, especially in CNVs, since the standard method to test for them is MLPA. Therefore, to make clinical decisions based on the identification of CNVs, confirmation with this technique is recommended [25]. On the other hand, the chosen panel included 50 genes, but the literature supports the notion that more actionable variants are identified the greater the panel is [30]. Lastly, the inclusion of patients depended on the clinical assessment of the medical oncologist, as patients whose evolution was unfavorable according to clinical criteria were analyzed; nevertheless, national and international protocols and clinical guidelines have been followed at all times, for both classification and treatment, as well as for the evaluation of the disease of the patients included, so the study uses a sample that represents a reproducible range of cases that can be found in clinical practice in other centers; however this fact lead to a relatively low sample size. Regarding the strengths of this project, the finding of genetic alterations in all samples should be highlighted. According to the literature and the approvals of targeted drugs, some alterations could have had therapeutic implications, and those that had prognostic implications could have prevented failed or ineffective treatments, which could in turn help in planning therapeutic strategies or favoring the inclusion of carriers in clinical trials. These observations should be understood, however, as hypothesis, yielding clues to be validated in prospective studies.

Taken together, for the sake of correct therapy optimization, the results presented in this study support the incorporation of NGS in the molecular diagnosis of patients with BC.

## Conclusions

According to conventional guidelines, recurrence occurs in an unacceptable percentage of cases, making imperative the search for new strategies. NGS sequencing of FFPE samples enables fast and efficient analysis of specific molecular alterations. In our study all patients with shortened progression-free survival or relapse-free survival presented with genomic alterations that could be identified by NGS, showing that it is a feasible and worthwhile technique in clinical practice. Furthermore, this approach can positively influence the management of this type of patient for oncologists as genomic alterations could be used as prognostic, predictive biomarkers or targets for therapies, rendering precision oncology effective.
